# Intraoperative Dexmedetomidine-Induced Polyuric Syndrome

**DOI:** 10.7759/cureus.1218

**Published:** 2017-05-03

**Authors:** Shannon Granger, David Ninan

**Affiliations:** 1 Anesthesiology, Riverside University Health System Medical Center, Moreno Valley, California, United States

**Keywords:** hypernatremia, polyuric syndrome, dexmedetomidine, diabetes insipidus

## Abstract

A 23-year-old male trauma patient with a cervical spine fracture underwent an anterior and posterior discectomy and spinal fusion surgery. The patient presented to the operating room with a stabilizing halo fixation device in place, and a fiberoptic intubation was performed with dexmedetomidine for sedation. During the surgical procedure, general anesthesia was maintained with a propofol and remifentanil infusion as the patient was monitored using somatosensory and motor evoked potentials. The patient's urine output increased gradually during the nine-hour surgical procedure from 150 mL/hour to over 700 mL/hour in the eighth hour of the procedure, where it remained until the end of the procedure. Postoperatively, the patient's laboratory values and urine output returned to baseline levels the following day. A search of the literature revealed few case reports of polyuria under similar conditions. Dexmedetomidine, being an alpha-2 agonist that blocks arginine-vasopressin release, may be responsible for inducing the polyuria noted in this patient case.

## Introduction

Dexmedetomidine is a medication that is frequently used for sedation in procedures such as fiberoptic intubation. It allows the patient to tolerate intubation while continuing to breathe spontaneously [1-2]. As an alpha-2 agonist that also blocks arginine-vasopressin release, polyuria is a theoretical side effect [3].

## Case presentation

A 23-year-old African American male presented two months after an injury from a motor vehicle collision where he sustained bilateral femoral fractures and a cervical spine injury. The patient had undergone a prior occipital to cervical level five posterior fusion and bilateral femur fracture fixation under general anesthesia without anesthetic complication. Other than the trauma, he had no significant past medical history. The patient’s family history was not significant for cancer, hypertension, endocrine, neurologic, or renal issues. He also reported he had no history of alcohol, smoking, and drug use. The patient was not currently taking any medication and reported he had no drug allergies.

On the day of surgery, the patient presented with a stabilizing halo fixation device in place and underwent an anterior cervical discectomy and fusion of cervical levels five through seven and a posterior spinal fusion of cervical levels five through thoracic level two.

The patient was premedicated with 2 mg intravenous midazolam. All standard American Society of Anesthesiologists-required monitors were placed, and an infusion of dexmedetomidine was initiated at 1 mcg/kg for 10 minutes as a loading dose, per dexmedetomidine drug protocol. After 10 minutes, the infusion rate was decreased to 1 mcg/kg/hour. The dexmedetomidine infusion was then stopped after intubation, and general anesthesia was initiated with a propofol drip at 100 mcg/kg/minute, and a remifentanil drip was initiated at 0.3 mcg/kg/minute; both drips were continued throughout the procedure as a general anesthetic. A bispectral index monitor was placed to monitor sedation levels. The anterior portion of the operation took approximately four hours, after which the patient was moved prone with appropriate spine stabilization.

The patient’s urine output was 150 mL one hour into the procedure. The patient’s urine output began increasing in the second hour until the completion of the nine-hour, 20-minute operation, reaching 750 mL/hour. During the surgical procedure, the patient received maintenance fluid as well as fluid to replace a presurgical deficit and blood loss, totaling 4.5 L of crystalloid. Blood loss was measured at 200 mL, and total urine output by the end of the procedure had reached 3065 mL (Table 1).

**Table 1:** Hourly polyuric workup during spinal surgery BP: Blood pressure; HR: Heart rate; Na: Sodium; Osm: Osmolality; Postop: Postoperative period; Preop: Preoperative period; UOP: Urine output.

		Surgical Procedure Hour									
Polyuric Workup	Preop	1	2	3	4	5	6	7	8	9	Postop
Serum Na (mmol/L)	136				148					149	145
Serum Osm (mOsm/kg)											307
Urine Na					15				39		108
Urine Osm					294				127		773
UOP (mL)		150	200	265	350	350	500	250	250	750	
HR (bpm)		50	60	55	55	65	70	75	65	60	
BP (mmHg)		100/60	100/60	100/50	110/60	110/60	100/50	100/60	100/60	110/60	
Blood Loss (mL)		0	20	20	20	40	40	20	20	20	
Fluid Administration (mL)		750	600	600	300	250	300	300	200	200	

The patient was hemodynamically stable throughout the procedure without significant episodes of hypotension or tachycardia, with a mean arterial pressure steady at approximately 65 mmHg. Due to the patient’s unexplained polyuria, a polyuric workup was initiated including urine and serum electrolytes and osmolalities. The patient’s initial serum sodium was 136 mmol/L, which was measured four days prior to the surgery. During the procedure, his sodium increased to 148 mmol/L, and, one hour following surgery, his serum sodium was 149 mmol/L. The patient’s intraoperative serum osmolality was within normal limits (294 mOsm/kg). However, his urine osmolality was as low as 55 mOsm/kg, and upon subsequent lab analysis, it increased to 127 mOsm/kg. The patient was extubated following a return to supine position, and he was monitored in the postanesthesia care unit for neurologic changes. The patient was then followed on the medical surgical unit. The patient’s urine output and laboratory values returned to baseline levels the following morning, and the patient was discharged home the following day without sequelae.

## Discussion

Polyuria is defined as a urinary output of greater than 3 L/day. Polyuria results from two mechanisms: (1) excretion of nonabsorbable solutes (e.g., glucose) or (2) excretion of water, usually from a defect in antidiuretic hormone production or renal responsiveness. Urine osmolality was measured to help distinguish the cause of polyuria. If urinary output is greater than 3 L/day with urine that is dilute (i.e., less than 250 mOsm/L) and the total mOsm excretion is normal then a water diuresis is present [3]. This comes from polydipsia (i.e., the inadequate secretion of vasopressin as seen in central diabetes insipidus) or failure of the renal tubules to respond to vasopressin (e.g., nephrogenic diabetes insipidus) [3].

As this case progressed, we searched for causal factors for the patient’s polyuria. Drugs other than dexmedetomidine administered during the case were midazolam, benzocaine topical spray, propofol, remifentanil, and fentanyl. We searched Lexicomp Online, and none of these medications were associated with polyuria. In contrast, opioids such as fentanyl and remifentanil have been shown to cause urinary retention. Further, our patient was not on any preoperative medications such as diuretics which would explain the polyuria. The patient had no prior history of polyuria and no confounding history of endocrine abnormalities like Cushing’s syndrome, Addison’s disease, or paraneoplastic syndromes. During the operation, his blood glucose was maintained at less than 180 mg/dL, ruling out hyperglycemia as a cause of the diuresis. No aberrant cardiac rhythms such as supraventricular tachycardia or atrial fibrillation were identified perioperatively, as evidenced by cardiac monitoring maintained throughout the surgery or postoperatively via electrocardiogram.

A literature review with the search terms “intraoperative polyuria” suggested that dexmedetomidine may have been a causal factor. Studies performed on canines undergoing general anesthesia have shown that dexmedetomidine has a diuretic-like effect associated with a decreased urine osmolarity, increased free water clearance, and a reduction in plasma vasopressin levels. These studies suggest that dexmedetomidine may induce both vasopressin suppression and responsiveness [4-5]. In humans, it is currently unknown if dexmedetomidine causes an inadequate secretion of vasopressin (a central mechanism) or if it decreases the responsiveness of the renal tubules to vasopressin (a peripheral cause).

Had dexmedetomidine been initially considered a contributing cause, a serum vasopressin level should have been measured to assess whether the mechanism was central or peripheral. However, plasma vasopressin levels were not measured in this patient, as dexmedetomidine was not elicited as a plausible cause until the end of the case.

Despite its widespread use, a literature search revealed four case reports of possible dexmedetomidine-induced polyuria. Pratt, et al. reported a medical case in which a patient received a multiple day infusion of dexmedetomidine for sedation [6]. The other three reported cases involve patient from spine surgery with an age range from 12 to 71 years [7-9]. The doses used in these cases were less than 1 mcg/kg/hour, and none of these cases used a loading dose. Once dexmedetomidine was discontinued in these cases, urine outputs normalized within two hours. Our case is distinct in that there was a loading dose and a short infusion during anesthesia induction, and the polyuria persisted well beyond the two-hour limit in the previously reported cases. This suggests that bolus administration may have a prolonged impact on dexmedetomidine-induced polyuria.

## Conclusions

In this case the administering dexmedetomidine as a bolus appeared to result in a longer lasting polyuria than reported in prior case studies. This suggests that even short-term use of dexmedetomidine can either suppress vasopressin levels or decrease renal responsiveness resulting in polyuria hours after discontinuation of the drug. Additionally, this case illustrates the need for further clinical studies on the diuretic effect of dexmedetomidine, which is especially important in patient populations where polyuria may exacerbate other comorbidities.
